# Position Effect Variegation of *Penicillium rubens* Genome and Application in Heterologous Production of the Plant‐Derived Anthraquinone Physcion

**DOI:** 10.1111/1751-7915.70449

**Published:** 2026-10-08

**Authors:** Meng Wu, Yushi Fu, Linqin Tang, Yongpeng Yao, Yuanyuan Pan, Gang Liu

**Affiliations:** ^1^ State Key Laboratory of Microbial Diversity and Innovative Utilization, Institute of Microbiology Chinese Academy of Sciences Beijing People's Republic of China; ^2^ University of Chinese Academy of Sciences Beijing People's Republic of China

**Keywords:** chromosomal position effect, filamentous fungi, heterologous biosynthesis, physcion, *PiggyBac*

## Abstract

The filamentous fungus *Penicillium rubens* is well known as an industrial host for producing penicillin and also used as a promising chassis for secondary metabolite biosynthesis. Chromosomal position effect significantly influences the functional implementation of exogenous genetic modules, but it remains underexplored in filamentous fungi such as *P. rubens* . In this study, a *PiggyBac* (*PB*) transposon‐mediated mutagenesis library was constructed in *P. rubens* . High‐throughput sequencing confirmed a broadly distributed with moderate local hopping of *PB* insertions across this fungal genome. To characterize the genome position effect, two genomic loci libraries including low‐expression (P4) and high‐expression (P7) positions were obtained respectively by flow cytometry. Heterologous biosynthesis of the plant‐derived anthraquinone physcion in *P. rubens* , via targeted integration of its biosynthetic gene cluster (BGC) into the P7 genomic loci, substantially boosted the titre to a level significantly higher than that achieved via integration at the P4 loci, demonstrating a direct correlation between the integration locus and the secondary metabolite output. To our knowledge, this is the first systematic elucidation of chromosomal position effect on heterologous biosynthesis of secondary metabolites in *P. rubens* and it also provides an insight for rational selection of integration sites in filamentous fungi.

## Graphical abstract

This study constructed a *PiggyBac* transposon mediated mutagenesis system which was used to investigate the genomic position effect on heterologous production of physcion in *Penicillium ruben*. This PB platform enables efficient genetic engineering for secondary metabolite production.

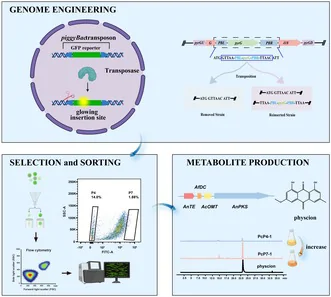

## 1 Introduction

*Penicillium rubens* (formerly referred to as *Penicillium chrysogenum*), a renowned producer of β‐lactam antibiotics and diverse secondary metabolites (e.g., roquefortine C and chrysogine) (Ding et al. 2020; Sawant et al. 2023), has emerged as a promising chassis for secondary metabolite production. Genomic sequencing revealed *P. rubens* harbours numerous the secondary metabolite biosynthetic gene clusters (BGCs) located at different genomic loci. However, most of them remain transcriptionally silent under the standard culture conditions (Van den Berg et al. 2008). Over the past decade, breakthroughs in the development of the genetic tools have revolutionized genome engineering in this species, thereby facilitating the heterologous expression of BGCs (Pohl et al. 2016).

Position effect variegation, driven by local chromatin structure (e.g., epigenetic marks and chromosome organization), critically influences the transcriptional activity of genes across eukaryotes (Yankulov 2013). In *Drosophila melanogaster* , translocation of the white gene near heterochromatin disrupted the eye pigmentation through position effect variegation (Henikoff 1990). In *Saccharomyces cerevisiae* , integration of exogenous genes into the silent chromatin region led to transcriptional repression (Schnell and Rine 1986). Similarly, the studies in mammal demonstrated that the euchromatin‐associated integration enhanced transgene expression compared to the heterochromatic loci. These findings underscore the universal role of chromatin microenvironments in modulating gene expression. However, the impact of chromosomal integration sites termed the “position effect” on heterologous gene expression remains underexplored in *P. rubens* (Palmer and Keller 2010). This knowledge gap persists owing to lack of high‐throughput tools for systematically analysing genome position effect in this filamentous fungus (Bilyk et al. 2017). Thus, there is an urgent need to develop chromatin‐aware engineering strategies in filamentous fungal hosts (Li et al. 2022).

To date, genome‐scale insertion libraries in filamentous fungi have been constructed primarily using a limited set of transposons, notably the *impala* element from *Fusarium oxysporum* (Langin et al. 1995). In *Aspergillus fumigatus* , *impala*‐based systems have enabled the generation of heterozygous diploid mutant libraries and the parasexual identification of essential genes (Firon et al. 2003; Carr et al. 2010). In addition, a transposon‐tagging method based on the endogenous *Vader* transposon has been developed in *Aspergillus niger* (Hihlal et al. 2011). Nevertheless, these approaches exhibit inherent limitations, including non‐random integration preferences that result in a high frequency of insertions within non‐coding regions and a tendency for local hopping, both of which reduce mutagenesis efficiency and hinder large‐scale saturation screening (Dufresne et al. 2008). Moreover, although low‐temperature‐inducible transposition has been demonstrated for the *impala* system (Carr et al. 2010), a broadly applicable, highly efficient and genuinely random mutagenesis platform remains absent for most industrially and medically significant filamentous fungi.

The *PiggyBac* (*PB*) transposon system, originally isolated from *Trichoplusia ni* , enables precise “cut‐and‐paste” integration into the TTAA sites without leaving genomic scars. Owing to this advantage, *PB* has been widely used in zebrafish, yeast and mammalian cells (Handler and Harrell 1999; Ding et al. 2005; Lobo et al. 2006). Notably, *PB* exhibits minimal integration bias in *Schizosaccharomyces pombe* and *Pichia pastoris*, achieving near‐random genome‐wide distribution which has been validated by deep sequencing (Li and Du 2014; Yao et al. 2026). This low positional preference, combined with its scarless excision capability, makes *PB* an ideal tool for unbiased exploration of chromatin‐dependent expression patterns (Li et al. 2011).

Despite these advantages, persistent challenges remain, such as unpredictable heterologous gene silencing due to integration into the repressive chromatin domains and the genomic instability caused by residual transposase activity. Although fluorescence‐activated cell sorting (FACS) of GFP‐tagged libraries has enabled the identification of expression “hotspots” in yeast, analogous strategies have not been systematically applied to *P. rubens* owing to the difficulty of manipulating multicellular filamentous microorganisms (Ongley et al. 2013).

Physcion, the 6‐O‐methylated derivative of emodin, is produced by several globally recognized herbs and exhibits a variety of pharmacological activities including laxative, hepatoprotective, anti‐inflammatory, anti‐microbial, and anti‐proliferative effects. The biosynthetic pathway of physcion originates from malonyl‐CoA, which is sequentially condensed by a thioesterase (TE)‐deficient non‐reducing polyketide synthase (NR‐PKS) and subsequently cleaved by a standalone metallo‐β‐lactamase‐type thioesterase (MβL‐TE) to yield atrochrysone carboxylic acid. This intermediate undergoes spontaneous decarboxylation to form atrochrysone, which is then converted to emodin anthrone via spontaneous dehydration, followed by oxidation to generate the direct precursor emodin. Notably, the decarboxylation step, while occurring spontaneously, can be significantly enhanced by enzymatic catalysis via decarboxylases, thereby improving pathway flux towards emodin. The final and critical step involves methylation of the C‐6 hydroxyl group of emodin, which is exclusively catalysed by an O‐methyltransferase using S‐adenosylmethionine as the methyl donor to produce the plant‐derived anthraquinone physcion (Yao et al. 2023). De novo biosynthesis of physcion has been achieved by introducing its BGC in *Aspergillus nidulans* and *Aspergillus terreus* (Qi et al. 2022; Yao et al. 2023), but the position effect on BGC expression has not been studied.

In this study, a *PB*‐based screening platform was developed to dissect position effect variegation in *P. rubens* . Using GFP as a reporter, a random insertion mutagenesis library was generated via *PB* transposition and subjected to FACS, yielding low‐expression (P4) and high‐expression (P7) populations with > 10‐fold difference in fluorescence. Integration site mapping revealed chromatin accessibility as a key determinant of gene expression levels, mirroring the eukaryotic “euchromatin activation” principle. Functional validation confirmed that strains with the biosynthetic gene cluster (BGC) integrated into loci from the P7 library showed substantially higher physcion production than those carrying the BGC inserted at loci from the P4 library. Finally, application of this platform enabled high production of physcion through targeted integration of the BGC into the transcriptionally active loci. To our knowledge, this is the first systematic elucidation of position effect on heterologous production of secondary metabolites in *P. rubens* . Our work indicates that *P. rubens* is a chromatin‐optimized chassis for secondary metabolite production and sets a precedent for rational integration‐site selection in filamentous fungi.

## 2 Experimental Procedures

### 2.1 General Experimental Methods

PCR was performed using *EasyTaq* DNA Polymerase from TransGen Biotech Co. Ltd. (Beijing, China) or Super‐Fidelity DNA Polymerase from Vazyme (Nanjing, China). DNA assembly was performed using ClonExpress II One Step Cloning Kit from Vazyme (Nanjing, China). All restriction enzymes and ligase were purchased from New England Biolabs (USA). Primer synthesis and DNA sequencing were conducted by Tsingke Biotech Co. Ltd. (Beijing, China). Plasmids were extracted by TIANprep Mini Plasmid Kit from Tiangen Biotech (Beijing, China), and DNA was purified by FastPure Gel DNA Extraction Mini Kit from Vazyme (Nanjing, China). High‐performance liquid chromatography (HPLC) was carried out on the SHIMADZU LC20‐AT system using a C18 analytical column (Hypersil GOLD 250 × 4.6 mm, 5 μm, Thermo) at a flow rate of 1 mL/min.

### 2.2 Strains, Media and Growth Conditions

The industrial fungus *P. rubens* Wisconsin 54‐1255 (formerly referred to *P. chrysogenum* Wisconsin 54‐1255) was used as the parent strain in this study. All fungal strains were grown on Potato Dextrose Agar (PDA) for 4 days at 28°C and their spores were collected. Czapek minimal medium (30 g/L sucrose, 2 g/L NaNO_3_, 0.5 g/L K_2_HPO_4_, 0.5 g/L MgSO_4_.7H_2_O, 0.01 g/L FeSO_4_) was used in fungal phenotype assays. 5‐Fluoroorotic acid monohydrate (5‐FOA) was used at a concentration of 1 mg/mL in the Czapek minimal medium supplemented with 100 μg/mL of uridine. All strains constructed in this work are listed in Table S1. All plasmids used in this work are listed in Table S2. The primers used in this work are listed in Table S3. PM medium (40 g/L glucose, 3 g/L NaNO_3_, 1 g/L KCl, 0.5 g/L K_2_HPO_4_, 0.5 g/L MgSO_4_.7H2O, 0.01 g/L FeSO_4_) was used for cultivation of *P. rubens* . PM‐S medium (20 g/L Starch, 3 g/L NaNO_3_, 1 g/L KCl, 0.5 g/L K_2_HPO_4_, 0.5 g/L MgSO_4_.7H_2_O, 0.01 g/L FeSO_4_) was used for heterologous production of physcion in *P. rubens* . *Escherichia coli* DH5α was used for routine plasmid construction and grown in the LB medium (10 g/L tryptone, 10 g/L NaCl and 5 g/L yeast extract) supplemented with required antibiotics, such as ampicillin (100 μg/mL).

### 2.3 Plasmids and Strains Construction

The genomic DNA of *P. rubens* was extracted as previously reported (Fierro et al. 2006). The G418 resistance cassette was amplified from pAg1PT‐G418 and combined in the *Sma*I site of the plasmid pUC19‐AMA1 via Gibson assembly to give pAG (Zhang et al. 2016; Liu et al. 2018). The *Cas9* expression cassette was amplified from pLC1 and cloned into the *Bam*HI site of pAG via restriction enzyme digestion and ligation to generate pACG (Chen et al. 2020). The gRNAs targeting the fungal genome were designed using sgRNAcas9 software according to the *P. rubens* Wisconsin 54‐1255 genome sequence (Van den Berg et al. 2008; Xie et al. 2014).

To construct the plasmid for *hyPBase* expression, the promoter P*gpdA* (GenBank: Z32524.1) was amplified from *A. nidulans* and the promoter P*glaA* (GenBank: Pc16g00620) was amplified from *P. rubens*, the codon‐optimized gene coding the hyperactive transposase (hyPBase) (Yusa et al. 2011) was synthesized by GenScript (NanJing, China) and the nourseothricin resistance gene (ZSJS) was amplified from pCSN44.NATS. The above fragments were incorporated into the pUC19‐AMA1 vector to generate the plasmid phyPBase‐P_gpdA_ and phyPBase‐P_glaA_.

For construction of pUC19‐LaeA, the *LaeA* fragment (GenBank: EU685842.1) amplified from *P. rubens* was linked to the *Eco*RI and *Kpn*I sites of pUC19 by Gibson assembly. For construction of pUC19‐PB, the *PBL* and *PBR* fragments amplified by PCR with pPB[ura4] as template were linked to the *Eco*RI and *Kpn*I sites of pUC19 by Gibson assembly (Li et al. 2011).

For constructing the strain PcWG001, the promoter of tRNA‐Leu was amplified from *P. rubens* with the primers Leu‐F/pyrg‐Leu‐1R. The sgRNA was amplified from the plasmid pLC2 with the primers pyrg‐gRNA‐1F/gRNA‐R (Chen et al. 2020). Next, the promoter of tRNA‐Leu and sgRNA were ligated into the *Pac*I site of pACG via Gibson assembly, generating pACG‐pyrg. The upstream and downstream homology arms of the *pyrG* gene were amplified from *P. rubens* with the primers p63‐1 U‐F/p56‐1 U‐R and p56‐3D‐F/p63‐1 U‐R respectively. The G418 resistance cassette was amplified from pAG with the primers p56‐2G‐F/R. Then, the upstream homology arm, the G418 resistance cassette and the downstream homology arm were ligated into the *Eco*RI and *Kpn*I sites of pUC19 via Gibson assembly to generate p19‐pyrgG418, which was subsequently introduced into *P. rubens* , and the transformants were selected on the Czapek minimal medium supplemented with 1 mg/mL of 5‐FOA and 100 μg/mL of uridine. Following antibiotic‐free passage, the plasmid was removed in the transformants and generated the strain PcWG001.

For constructing the strain PcWG002, the upstream and downstream homology arms of the *G418* resistance gene were amplified from the genomic DNA of PcWG001 with the primers p63‐1 U‐F/p57‐1 U‐R and p57‐2D‐F/p63‐3D‐R respectively. The *PBL* and *PBR* fragments amplified by PCR with pPB[ura4] as template (Li et al. 2011), together with the *pyrG* (Gene ID: 8312314) cassette amplified by PCR with the genome DNA of *P. rubens* as template, were linked to the *Eco*RI and *Kpn*I sites of pUC19 to give the plasmid pPB‐pyrg. Then, the promoter P*gndA* (GeneID: 4984433) derived from *A. niger* , the *tef1* terminator (Yao et al. 2023) derived from *A. nidulans* , and the *GFP* gene were ligated to the *Pac*I site of pPB‐pyrg to generate the plasmid pPB‐pyrgG. Subsequently, pACG‐pyrg and pPB‐pyrgG were transformed into *P. rubens* to generate the strain PcWG002, which was selected on the Czapek minimal medium.

For constructing the *ku70* disruption mutant (ku70DM), the tRNA‐Leu promoter used to express the gRNA was amplified from *P. rubens* with the primers Leu‐F/ku70‐Leu‐1R. The sgRNA was amplified from pLC2 (Chen et al. 2020) with the primers ku70‐gRNA‐1F/gRNA‐R. The tRNA‐Leu promoter and sgRNA were subsequently ligated into the *Pac*I site of pACG via Gibson assembly, generating pACG‐ku70, which was subsequently introduced into *P. rubens* , and the transformants were selected on the Czapek minimal medium supplemented with 100 μg/mL of G418 after 3 days of cultivation.

For constructing the *pcbAB* disruption mutant, the promoter of tRNA‐Leu for expressing the gRNA was amplified from the genomic DNA of *P. rubens* with the primers Leu‐F/pcbab‐Leu‐1R. The sgRNA was amplified from the plasmid pLC2 (Chen et al. 2020) with the primers pcbab‐gRNA‐1F/gRNA‐R. Then the promoter of tRNA‐Leu and sgRNA were ligated into the *Pac*I site of pACG, generating pACG‐pcbAB. The DNA fragments corresponding to the upstream and downstream regions of *pcbAB* were amplified from *P. rubens* using the primers pcbABU‐F/pcbABU‐R and pcbABD‐F/pcbABD‐R, respectively, and ligated into the *Pac*I site of pACG‐pcbAB to generate pACG‐pcbABdonor. Subsequently, the plasmid pACG‐pcbABdonor was transformed into *P. rubens* to generate the *pcbAB* disruption mutant (pcbABDM).

### 2.4 Preparation of Protoplasts From *P. rubens*

Conidia of *P. rubens* (1 × 10^7^) were inoculated in 50 mL PM medium of a 250‐mL flask and incubated at 28°C, 250 rpm for 24 h. Subsequently, the fungal mycelia were collected by filtration on a nylon filter and washed with 0.9% NaCl. To obtain protoplasts, the mycelia were resuspended in 10 mL lytic buffer (1.2 M MgSO_4_, 10 mM sodium phosphate buffer) with 0.64 g enzyme (VINOTASTE PRO, Novozymes). The lytic mixture was incubated at 28°C with gentle shaking for 4 h, and purified gently and slowly by adding trapping buffer (0.6 M Sorbitol, 0.1 M Tris‐HCI, pH 7.0) followed by centrifugation at 4000 rpm for 20 min. The protoplasts collected from the interface were washed three times with 0.7 M KCl, collected, centrifuged at 4000 rpm for 2 min and resuspended in the KCM solution (0.7 M KCl, 50 mM CaCl_2_, 10 mM MOPS, pH 5.8).

### 2.5 Transformation of *P. rubens*

The *P. rubens* protoplasts were obtained and the transformation was performed as previously described (Cantoral et al. 1987). Then, the transformants were selected in the Czapek minimal medium supplemented with 1 M sorbitol and 100 μg/mL of G418 or 300 μg/mL of nourseothricin.

### 2.6 Transposition Induction and Genetic Screening of the *P. rubens* Strains

In the experiment for *PB*[*pyrG*] integration at the *G418* locus, the initial strain harbouring the plasmid for expressing the *PBase* gene was incubated in the Czapek minimal medium. The mycelia were then harvested with 1 mL sterile water, and 50 μL of the resulting mycelial suspension was transferred onto the PDA plates supplemented with 1.5% starch to induce *hyPBase* expression, and the transferred mycelia were allowed to continue growing on these induction plates throughout the subsequent incubation period. At the indicated time points, the conidia from the strains grown in PDA were collected, washed, and re‐suspended in ddH_2_O. To obtain the transposable strains with pyrG^+^ and G418^+^, the conidia suspension was diluted and spread on the Czapek minimal medium with 100 μg/mL of G418.

### 2.7 TAIL‐PCR for Mapping the *PB* Insertion Sites

The genomic DNA of *P. rubens* and its derivatives was isolated (Fierro et al. 2006). Thermal asymmetric interlaced PCR (TAIL‐PCR) assay was carried out using the genomic DNA of the transposable strains as templates as previously described (Liu and Chen 2007). The specific primer PBL‐a1 adjacent to the target flanking sequence was combined with two random primers (LAD1‐1 and LAD1‐3) with low Tm values and amplified by PCR with thermally asymmetric temperature cycling. The resulting PCR product was amplified again using the nested primer PBL‐a2 of PBL‐a1 and AC1 (Table S3) to obtain a flanking sequence. The PCR product was sequenced and compared with the genome sequence of *P. rubens* to identify the *PB* insertion sites.

### 2.8 Detecting the Copy Number of *PB* in *P. rubens*

The genomic DNA from the transposable strains was analysed on Mastercycle ep realplex equipment (Eppendorf, Germany) using SYBR Premix Ex TaqTM II PCR kit (TaKaRa, Dalian, China). PCR was carried out with primers RT‐PBL‐F/RT‐PBL‐R for amplifying the *PB* element, with primers RT‐LaeA‐F/RT‐LaeA‐R for amplifying *laeA*, which is a single copy in *P. rubens* Wisconsin 54‐1255 (Cantoral et al. 1987). The *PB* copy number was determined by the ΔΔCt method using genomic DNA from PcWG002, a strain carrying a single *PB* copy, as the control. The standard curve of absolute quantification was created by a seven orders of magnitude dilution series in triplicates of a water solution containing pUC19‐LaeA and pUC19‐PBL. For PCR, the diluted template DNA (0.8 μL), ddH_2_O (7.8 μL) and each of the primers (0.8 μL) were added to the PCR mixture (20 μL) and subjected to 40 cycles of PCR. Melting curve analysis after the cycling confirmed the absence of nonspecific products in the reaction. The fluorescence threshold (C_t_) values were calculated for standards and samples by using the realplex software. Because the ratio of *PB* to *laeA* was usually not an integer, we estimated the copy number to be the integral closest to the ratios.

### 2.9 Flow Cytometry

To improve protoplast preparation efficiency for flow cytometry analysis, conidia collected from the PDA plates supplemented with 1.5% starch were incubated in the liquid Czapek minimal medium with 100 μg/mL G418 at 28°C for 36 h. Subsequently, the collected mycelium was suspended in the lytic buffer and incubated at 28°C with gentle shaking for 4 h, and purified with trapping buffer. The protoplasts collected from the interface were washed and re‐suspended with 0.7 M KCl, then recovered by filtration on a nylon filter. Protoplasts were analysed by using BD FACS Calibur Flow Cytometer with a 488 nm excitation laser and an FL1 (530/30 nm band‐pass filter) detector. Cells were first gated on FSC‐A vs. SSC‐A to exclude debris, followed by doublet discrimination using FSC‐W/FSC‐H and SSC‐W/SSC‐H. Autofluorescence was corrected using an untransformed parental strain processed in parallel, and GFP signal was recorded as the difference from the control. Based on the fluorescence histogram, the P4 gate (GFP fluorescence consistent with negative control) comprised 14.0% of the total population, while the P7 gate (highest GFP fluorescence) comprised the top 1.88%.

### 2.10 Mapping the *PB* Insertion Sites by High‐Throughput Sequencing

The conidia of *P. rubens* were collected upon transposition induction at 28°C for 4 days on the PDA supplemented with 1.5% starch and incubated in the liquid Czapek minimal medium supplemented with 100 μg/mL of G418. Genomic DNA was isolated as previously described (Fierro et al. 2006). Tagmentation was carried out by using the TruePrep DNA Library Prep Kit V2 for Illumina (Vazyme Biotech, TD501) according to the manufacturer's instructions. The first round of PCR was carried out to amplify the *PB* element together with its flanking sequences using a *PB*‐specific primer close to the insertion site, PBLseq, and a primer annealing to the Tn5 adaptor, N708. The cycling parameters were: 3 min at 72°C; 30 s at 98°C; 12 cycles of 15 s at 98°C, 30 s at 60°C and 3 min at 72°C; 10 min at 72°C; 4°C store. The second round of PCR was aimed to add sequences required for Illumina sequencing with an Illumina flowcell attachment primer, DM508 (Vazyme Biotech, N321), and partial Tn5 adaptor primer, Illumina‐R. Cycling conditions are: 1 min at 98°C; 7 cycles of 10 s at 98°C, 75 s at 67°C; 5 min at 67°C; 4°C store. Sequencing data were analysed as previously described (Li et al. 2020), with the modifications described below. The reference genome sequence can be obtained under accession number JBYQAM000000000. The in‐house scripts are publicly available in GitHub at https://github.com/xchromosome219/PBseq.pipeline under an MIT licence.

### 2.11 Hi‐C Library Construction and Data Processing

The Hi‐C library was constructed using the Hi‐C 2.0 protocol with *Mbo*I and sequenced on the BGI PE150 platform. For the parental strain *P. rubens* Wisconsin 54‐1255, 452.8 million paired‐end reads were generated for each biological replicate. After trimming adapters and filtering low‐quality reads using Trimmomatic, clean reads were aligned to the reference genome using BWA‐MEM with the ‐SP parameter. The resulting SAM files were processed with pairtools to filter valid interaction pairs, retaining only uniquely mapped, non‐self‐ligated pairs (Cheng et al. 2022).

The genome was partitioned into consecutive, non‐overlapping bins of 10 kb for downstream analysis. An initial contact matrix was built by assigning valid pairs to their corresponding bins. To correct for technical biases, the matrix was normalized using the Iterative Correction and Eigenvalue decomposition (ICE) method. The effective resolution was defined as the highest resolution at which at least 80% of bins contained ≥ 1000 contacts (Rao et al. 2014).

To define genomic compartments, we applied the cooltools (PCA method) on the 10‐kb normalized contact matrix. Bins with positive or negative PC1 values were assigned to Compartment A or B, respectively. For the identification of topologically associating domains (TADs), we employed the insulation score method as implemented in the cworld‐dekker pipeline, with a window size of 10× resolution. TAD boundaries were defined as regions with locally minimal insulation scores (Crane et al. 2015).

To assess the enrichment of *PB* insertions from the P4 and P7 libraries in specific chromatin features, we performed Fisher's exact test. The significance of differences in gene expression levels between the P4 and P7 groups was determined using a two‐way ANOVA. For the Hi‐C data reproducibility, the concordance between biological replicates was evaluated using the GenomeDISCO method to generate a reproducibility score, as previously described (Cheng et al. 2022).

### 2.12 Heterologous Production of Physcion in *P. rubens*

The phy01 fragment containing the polyketide synthase encoding gene *AnPKS* was amplified from the plasmid pEP05 (Yao et al. 2023). Similarly, the phy02 fragment containing the thioesterase encoding gene *AnTE*, the decarboxylase encoding gene *AfDC*, and the O‐methyltransferase encoding gene *AcOMT* was amplified from the plasmid pEP11 (Yao et al. 2023). To construct the strains for producing physcion, the DNA fragments corresponding to the upstream and the downstream regions of P4‐1, respectively, were amplified from *P. rubens* using the primers P4‐1U‐F/P4‐1U‐R and the primers P4‐1D‐F/P4‐1D‐R. The DNA fragment ZSJS containing the nourseothricin resistance gene was amplified from pCSN44. NATS. The above fragments were recombined to the *Eco*RI and *Kpn*I sites of pUC19 to generate p19‐P4‐1. Then, phy01 and phy02 were recombined to the *Pac*I sites of p19‐P4‐1 to generate pP4‐1P. Meanwhile, the cassettes for targeting P4‐1 were amplified from pACG‐pcbAB with the corresponding overlapped primers and ligated into the *Pac*I site of pACG, generating pACG‐P4‐1. Subsequently, pP4‐1P and pACG‐P4‐1 were transformed into *P. rubens* to generate strain PcP4‐1. Similarly, strains PcP4‐1, PcP4‐2, PcP4‐3, PcP4‐4, PcP4‐5, PcP7‐1, PcP7‐2, PcP7‐3, PcP7‐4 and PcP7‐5 were generated through homologous recombination of phy01 and phy02 into their respective chromosomal loci.

To detect the physcion (CAS: 521‐61‐9) production, 1 × 10^6^ spores from each strain were inoculated into the PM‐S medium for 3 days at 28°C. The fermentation culture was then subjected to ultrasonic extraction using an equal volume of methylene chloride for 30 min, with the extraction process repeated three times. The resultant extract was subsequently dissolved in 300 μL methanol for high‐performance liquid chromatography (HPLC) analysis. HPLC analysis was performed with a linear gradient of 10%–100% acetonitrile (MeCN)‐H_2_O (v/v, 0.1% formic acid) for 25 min followed by 100% MeCN (v/v, 0.1% formic acid) for 5 min and rebalanced by 10% MeCN (v/v, 0.1% formic acid) for 8 min with a flow rate of 1.0 mL/min.

### 2.13 RNA Isolation and Real‐Time RT‐PCR Analysis

The mycelia of *P. rubens* cultured on the modified PM‐S medium (specifically optimized for physcion production) were harvested after 3 days of growth. Total RNA extraction and quantitative real‐time RT‐PCR were performed following the established protocols (Li et al. 2013).

### 2.14 Statistical Analysis

Quantitative data are presented as mean ± SD unless otherwise indicated. Independent biological replicates were used for statistical analyses, and technical replicates were averaged before analysis where applicable. Statistical analyses were performed using (software and version). Comparisons between two independent groups were performed using two‐sided Student's *t*‐tests where appropriate. Comparisons involving more than two groups were performed using one‐way or two‐way ANOVA followed by Tukey's multiple‐comparison test, as specified in the corresponding figure legends. For Hi‐C‐based enrichment analyses, 10,000‐sample permutation tests were performed using the genome‐wide PB insertion sites as the background. For comparisons of distances to TAD boundaries, two‐sided Wilcoxon rank‐sum tests were used. Compartment distributions were compared using the χ^2^ test. Benjamini–Hochberg correction was applied to account for multiple comparisons where appropriate. Exact *p‐*values are reported where applicable, and individual biological replicate values are shown in the corresponding figures.

## 3 Results

### 3.1 Transposition Using the Modified *piggyBac* System in *P. rubens*

To test the feasibility of *piggyBac* (*PB*) in *P. rubens* , we developed a binary transposition system. Within this system, a strain was constructed and designated as PcWG001 (Figure 1A), in which the orotidine 5′‐phosphate decarboxylase gene *pyrG* was replaced by a G418 resistance cassette. The transposon was constructed to generate the donor plasmid pPB[pyrG] which contained *pyrG* under the control of the *TrpC* promoter and the *TrpC* terminator and flanked by the inverted terminal repeats of *PB*. Subsequently, we inserted the PB[pyrG] cassette immediately after the initiation codon ATG within the open reading frame of the G418 resistance gene, resulting in the strain PcWG002 (Figure 1A), which exhibited sensitivity to G418 and uridine prototrophy. The codon‐optimized gene *hyPBase* encoding the hyperactive transposase, under the control of the constitutive promoter of P*gpdA* from *A. nidulans* , was expressed based on a plasmid containing autonomous replication sequence AMA1 (Yusa et al. 2011). Additionally, a nuclear localization signal (NLS) from SV40 was appended to its N‐terminus and a NLS from nucleoplasmin was added to its C‐terminus (Figure 1A).

**FIGURE 1:**
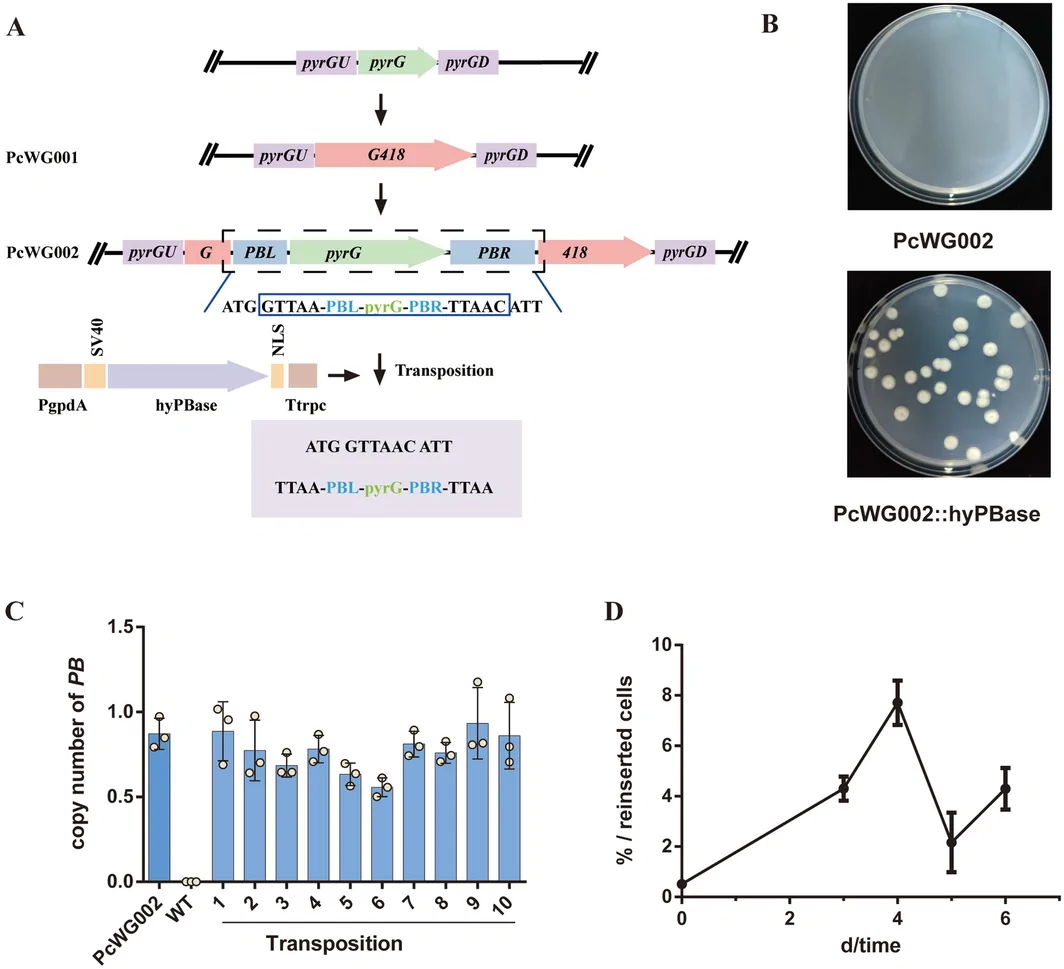
*PB* transposition in *P. rubens* . (A) Schematic description of the PB[pyrG] cassette integrated within the G418 resistance gene in PcWG002. (B) Selection of the transformants with PB[pyrG] integration on the MM supplemented with G418. (C) Analysis of *PB* copy number in the transposable strains using qPCR. PcWG002 and WT were used as controls. Error bars represent s.d. from the mean of triplicate samples. (D) Estimation of transposition efficiency in PcWG002, defined as the percentage of G418‐resistant transformants among the total number of transformants. Efficiency = (number of colonies on MM + G418)/(number of colonies on MM medium). Error bars represent s.d. from the mean of three independent experiments.

To achieve *PB* transposition in *P. rubens* , an episomal plasmid carrying the *hyPBase* gene was transformed into PcWG002. The resulting strain was cultured on PDA for 5 days; the fungal spores were collected and spread on the G418‐containing PDA plates. Since the parent strain PcWG002 was sensitive to G418, only the cells regaining the G418 cassette through transposition could robustly grow on the Czapek minimal medium supplemented with 100 μg/mL of G418. As expected, expressing *hyPBase* in the transformants resulted in the proliferation of numerous colonies, whereas PcWG002 alone did not grow (Figure 1B). Meanwhile, only the transformants remaining the entire *pyrG* gene could grow on PDA. This outcome demonstrated that *hyPBase* was expressed and excised the *PB* cassette from the G418 cassette and subsequently re‐inserted it into another locus of the fungal genome. Moreover, *PB* excision did not leave any residual footprint, a finding that has been consistently observed across all host systems examined to date (Elick et al. 1996).

To investigate the reinsertion sites of *PB*, TAIL‐PCR was carried out. The resultant PCR products exhibited various sizes and different banding patterns (Figure S1), indicating that the *PB*‐flanking sequences were likely corresponded to the different regions within the *P. rubens* genome. Through DNA sequence analysis of the PCR products and homology alignment with the *P. rubens* genome, we successfully mapped the *PB* reinsertion sites to different loci. Among the 36 mapped reinsertion events, the sequence TTAA at the recognition sites did not exhibit a strong bias (Table S4). Notably, 22 transposition events occurred at the same site, Chromosome II (n.t. 2,833,126‐2,833,129), located 436 base pairs downstream of the donor locus. This pattern suggested that transposon had a preferential insertion bias towards regions near the donor locus, an effect that is widely known as local hopping. Advantageously, the *PB* transposon did not appear to strongly favour or avoid any particular regions of the *P. rubens* genome. Our results clearly demonstrated that the modified *PB* system could achieve scarless excision and random re‐insertion, providing an efficient strategy for constructing a random insertion mutagenesis library in *P. rubens* .

Simultaneous insertion of transposons at multiple genomic loci, which could complicate the identification of mutations responsible for specific phenotypes, is a common issue in mutagenesis studies. The *PB* system facilitates transposition from a chromosomal site using a single‐copy transposon. Nevertheless, even with a single‐copy transposon on the chromosome, the transposon copy number may increase when transposition occurs during the S phase or G2 phase of the cell cycle (Engels et al. 1990). Generally, one *PB* copy remains at the original donor site; thus, some daughter cells contain two copies of *PB*. Our screening system excluded the daughter cells containing two *PB* copies, and only strains that restored the G418 resistance grew in the G418‐containing medium. To investigate whether the *PB* copy number changes during transposition, the *PB* copy number in G418^+^ colonies was calculated using quantitative PCR. Following a 4‐day induction of the *hyPBase* gene expression by 1.5% starch on PDA, the spores were collected and spread on the Czapek minimal medium supplemented with G418. Finally, 10 G418^+^ colonies were randomly picked. Among the transposable strains examined, all colonies contained only one copy of the *PB* element (Figure 1C). This finding suggested that each of the transposable strains only harboured a single *PB* insertion. The observed variation in the reporter gene expression was attributable not to the copy number of the *PB* element, but rather to the insertion site.

Since the *hyPBase* gene was expressed under the control of a constitutive promoter P*gpdA*, which resulted in genetic instability of the transposable strains, we replaced the constitutive promoter with the starch‐inducible promoter P*glaA* from *P. rubens* . Given that *P. rubens* is a multicellular microorganism, the transposition efficiency of *PB* was assessed through an induction process conducted on the solid agar plates. The resulting spores were subsequently inoculated in the selective medium for statistical analysis. Specifically, the fungal cells were induced on the PDA supplemented with 1.5% starch and collected at various time points. Portions of the collected spores were then spread on the Czapek minimal medium, where all cells could grow. In the MM plates supplemented with G418, only the G418^+^ cells were permitted to grow. Thus, the transposition efficiency was calculated by dividing the number of colonies on the MM plates containing G418 by the total number of colonies on the Czapek minimal medium. The transposition efficiency exhibited a gradual increase over time, peaking on Day 4, followed by a subsequent decrease (Figure 1D), potentially caused by a second transposition event. The results demonstrated that the inducible promoter could effectively control *hyPBase* expression, although it should be noted that transposition events occasionally occurred even in the absence of the inducer.

### 3.2 Random Insertion of *PB* in the *P. rubens* Genome

To evaluate whether the *PB* transposon confers an advantage in constructing the random insertion mutagenesis library, we analysed the chromosomal distribution of *PB* in the *P. rubens* genome using high‐throughput sequencing. Transposition was induced by 1.5% starch on PDA for 4 days, after which the collected spores were cultured in the Czapek minimal medium supplemented with G418. This selective medium allowed growth of the transposable cells, while the parent strain failed to germinate owing to its sensitivity to G418. Subsequently, the germinated mycelium was lysed to produce protoplasts, from which the fungal genomic DNA was directly extracted. The flanking sequences of PBL were then processed into libraries for Illumina sequencing. A total of 13,059 independent insertions were identified and supported by uniquely aligned reads (Figure 2A). In previous studies on human cells, mice, *S. cerevisiae* and *C. albicans* , the majority of *PB* insertions predominantly targeted the TTAA sequence (Ding et al. 2005; Li et al. 2011; Gao et al. 2018). In contrast, our findings demonstrated a weak preference for the specific insertion sites at the primary DNA sequence level. Nucleotide motif analysis of the insertion sites revealed 40% at TTAA sites, and the majority of insertion sites lacked a conserved sequence (Figure 2B). This divergence suggests that *PB* transposition may exhibit species‐specific adaptations, potentially influenced by chromatin accessibility, epigenetic modifications or host factor interactions. We also analysed the sequences at the insertion junctions to reveal any peripheral preference. Some preferences were observed, such as G at the −3, −2 and −1 positions, which exhibited preferences totally different from those in *S. cerevisiae* , insect, mouse and *C. albicans* , in which *PB* has a slight preference for Ts in the five bases upstream of TTAA and As in the five bases downstream of TTAA (Li et al. 2011; Gao et al. 2018).

**FIGURE 2:**
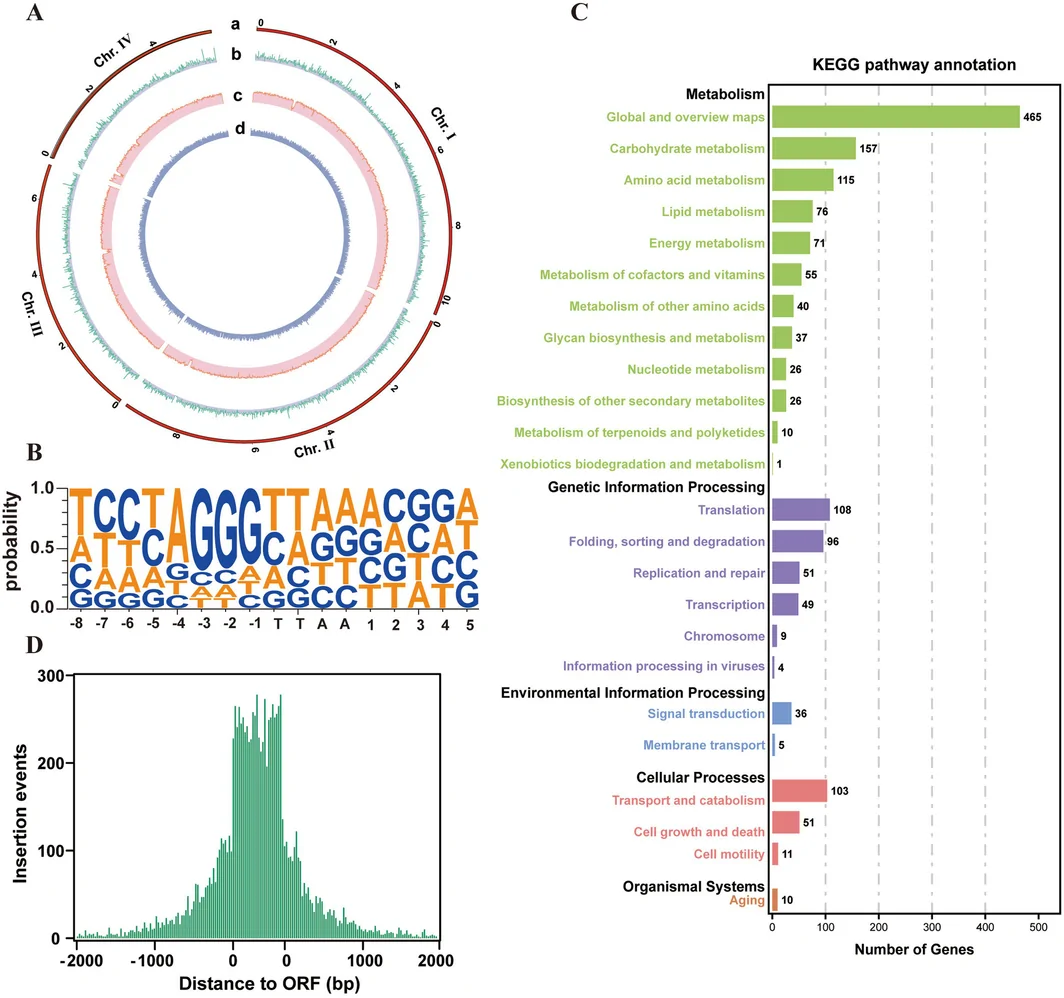
Distribution of the re‐inserted *PB* transposon in the *P. rubens* genome. (A) Genome‐wide NGS analysis of the *PB* distributions. The PB‐specific reads were plotted in 10‐kb sliding windows. a, Schematic representation of chromosomal size (2 Mb scale). b, Gene density represented by number of genes in 100‐kb non‐overlapping windows. c, Distribution of the GC content. d, Distribution of the *PB* transposon insertions on the *P. rubens* genome. (B) Motif analysis of the *PB* insertion sites. (C) Kyoto Encyclopedia of Genes and Genomes analysis of genes with *PB* insertion. (D) The *PB* insertion sites were plotted relative to ORF positions. Each ORF was divided into 25 equal‐sized segments and the number of insertions in each segment was displayed. X‐axis means distance to ORF while y‐axis represents integration events in vivo.

Subsequently, we analysed the distribution of *PB* insertions within 10 kb windows. Mapping of *PB* insertions across the *P. rubens* genome revealed a broadly distributed pattern (Figure 2A). There was a discernible yet moderate enrichment of *PB* insertion sites within a 100‐kb region surrounding the *pyrG* locus, indicating a mild local hopping effect. Overall, our data suggest that *PB* insertions do not show a pronounced preference or avoidance for specific regions of the *P. rubens* genome. The proportion of insertions within genes was 35.3%, while the proportion of insertions in intergenic regions was 64.7% (Figure S2A). KEGG annotation indicated that 30.7% of the 4609 genes with insertions were involved in metabolism, followed by genetic information processing (8.5%) and cellular processes (6.6%) (Figure 2C). AntiSMASH analysis identified 59 secondary metabolite biosynthetic gene clusters (BGCs) in *P. rubens*. The *PB* transposon insertions were detected across all of these BGCs. Notably, the core biosynthetic genes of 43 BGCs were disrupted by the *PB* insertions, suggesting that these clusters are not essential for fungal growth. Furthermore, the *PB* insertions were also found in additional biosynthetic genes, regulatory genes, transporter genes, as well as other genes associated with the clusters (Dataset S1). These insertion sites provided a foundation for the targeted genome minimization of *P. rubens* in the future. To survey the positional bias of the *PB* insertions, we plotted the distribution density of insertions in the intergenic regions (IGRs) and open reading frames (ORFs) (Figure 2D). In *S. cerevisiae* and *S. pombe*, peaks of insertions of transposons in IGRs were close to the 5′ or 3′ end of ORFs, which was believed to be caused by nucleosome occupancy in yeast (Gangadharan et al. 2010; Guo et al. 2013). However, our findings revealed that the *PB* transposon exhibited the highest insertion density within the ORFs, with a gradual decrease in density away from the ORFs. This preference facilitated gene mutagenesis, thereby aiding in the identification of genes of interest. Therefore, the PB transposon appears to be a suitable tool for constructing random insertion mutagenesis libraries.

### 3.3 Identification of the Loci With Different Expression Level

To systematically and comprehensively investigate the effect of genomic locus on heterogeneous gene expression, we constructed a strain carrying the PB system, with GFP used as a reporter. The PB[pyrG]‐GFP cassette was inserted immediately following the translation start codon ATG within the open reading frame of the G418 resistance gene, generating the strain PcWG003. In this strain, *GFP* expression was controlled by the moderately strong promoter P*gndA* derived from *Aspergillus niger* (Mózsik et al. 2019). Subsequently, the transposition plasmid phyPBase‐P_glaA_ was introduced into PcWG003 to generate the strain PcWG003P. Based on variations of the *GFP* expression level, single cells were sorted using flow cytometry as reported previously, in conjunction with high‐throughput sequencing technology, to construct the insertion mutagenesis libraries exhibiting different expression levels of *GFP* in *P. rubens* (Wei et al. 2022).

For sorting the single cell by flow cytometry, it was necessary to lyse the fungal mycelium to form single‐cell protoplasts. Using flow cytometry, single‐cell protoplasts were sorted based on *GFP* expression level. However, the fungal colonies on the solid plates deeply penetrated into the medium, hindering the acquisition of sufficient mycelium for enzymatic hydrolysis. Additionally, the mycelia grown on the solid plates were extremely resistant to enzymatic hydrolysis. Thus, we developed a high‐throughput liquid methodology for screening and separating spores and protoplasts (Figure S3). Protoplasts derived from the wild‐type (WT) strain were used as a negative control. Flow cytometry revealed subsets of protoplasts with varying expression levels of *GFP*, designated as P4 and P7 respectively, within the prepared protoplasts. P4 represented protoplasts consistent with the negative control, comprising 14.0% of the population, while P7 exhibited the highest *GFP* expression, accounting for 1.88% (Figure 3A). Genomic DNA was extracted from two distinct protoplast populations and subjected to next‐generation sequencing (Table S5). To further validate that the P4 and P7 populations represented stable low and high‐expression genomic loci rather than transient physiological states, five independent clones from each population were randomly selected for characterization. All tested clones retained their corresponding GFP fluorescence phenotypes, harboured a single *PB* insertion and maintained stable GFP expression after five generations of non‐selective propagation (Figure S4A and S4B).

**FIGURE 3:**
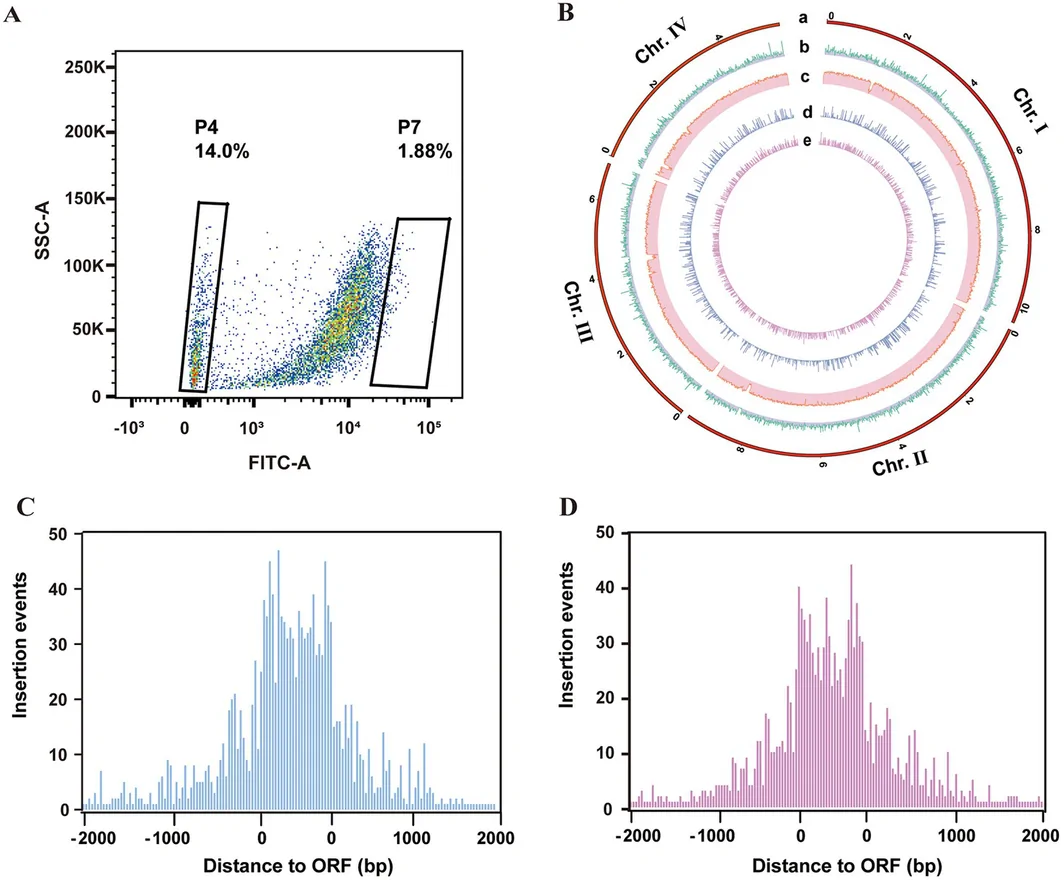
Construction and Characterization of the P4 and P7 libraries. (A) Workflow of single‐cell quantification for gene expression in *P. rubens* by flow cytometry. P4 and P7 denote the low‐ and high‐GFP‐fluorescence populations, respectively. (B) Genome‐wide analysis of the *PB* distribution in P4 and P7 libraries. a, Schematic representation of chromosomal size (2 Mb scale). b, Gene density represented by number of genes in 100‐kb nonoverlapping windows. c, Distribution of GC content. d, Distribution of the *PB* insertions on *P. rubens* genome in P4 library. e, Distribution of the *PB* insertions on *P. rubens* genome in P7 library. (C) The *PB* insertion sites in the P4 library were plotted relative to ORF positions. (D) The *PB* insertion sites in the P7 library were plotted relative to ORF positions. Each ORF was divided into 25 equal‐sized segments and the number of insertions in each segment was displayed. X‐axis means distance to ORF while y‐axis represents integration events in vivo.

In the P4 and P7 libraries, 1341 and 1222 independent insertion loci were identified respectively (Figure 3B), with insertions relative to the internal genes accounting for 54.81% (Figure S2B) and 52.86% (Figure S2C) of total events in each library. To characterize the insertion‐site preference across the P4 and P7 libraries, distribution density analysis of the *PB* insertions within IGRs and ORFs was performed (Figure 3C,D). Our findings revealed a slight but consistent preference for the *PB* insertion into ORFs, which was in agreement with observations from the genome‐wide insertion pattern analysis.

### 3.4 Structural Basis of Chromosomal Position Effect Variegation in *P. rubens*

To reveal the mechanism of the target gene expression variation at differential insertion loci from the P4 and P7 libraries respectively, high‐resolution Hi‐C was employed to map the three‐dimensional (3D) chromosomal architecture of the parent strain *P. rubens* Wisconsin 54‐1255. Hi‐C profiling revealed distinct compartments A and B characterized by positive and negative eigenvectors respectively, mirroring mammalian chromosomal organization where these compartments were correlated with transcriptionally active and inactive chromatin domains (Lieberman‐Aiden et al. 2009).

P7 insertion sites showed a modest but significant enrichment in compartment A (Figure 4A,B). However, P7 sites were underrepresented relative to the *PB* background. In contrast, P4 sites did not show significant enrichment in compartments A and B (Figure S5A). These results indicate that P7 high‐expression sites are associated with compartment A localization, whereas P4 low‐expression sites are not simply explained by compartment B enrichment, suggesting that additional local genomic or chromatin‐related features may contribute to the low‐expression phenotype.

**FIGURE 4:**
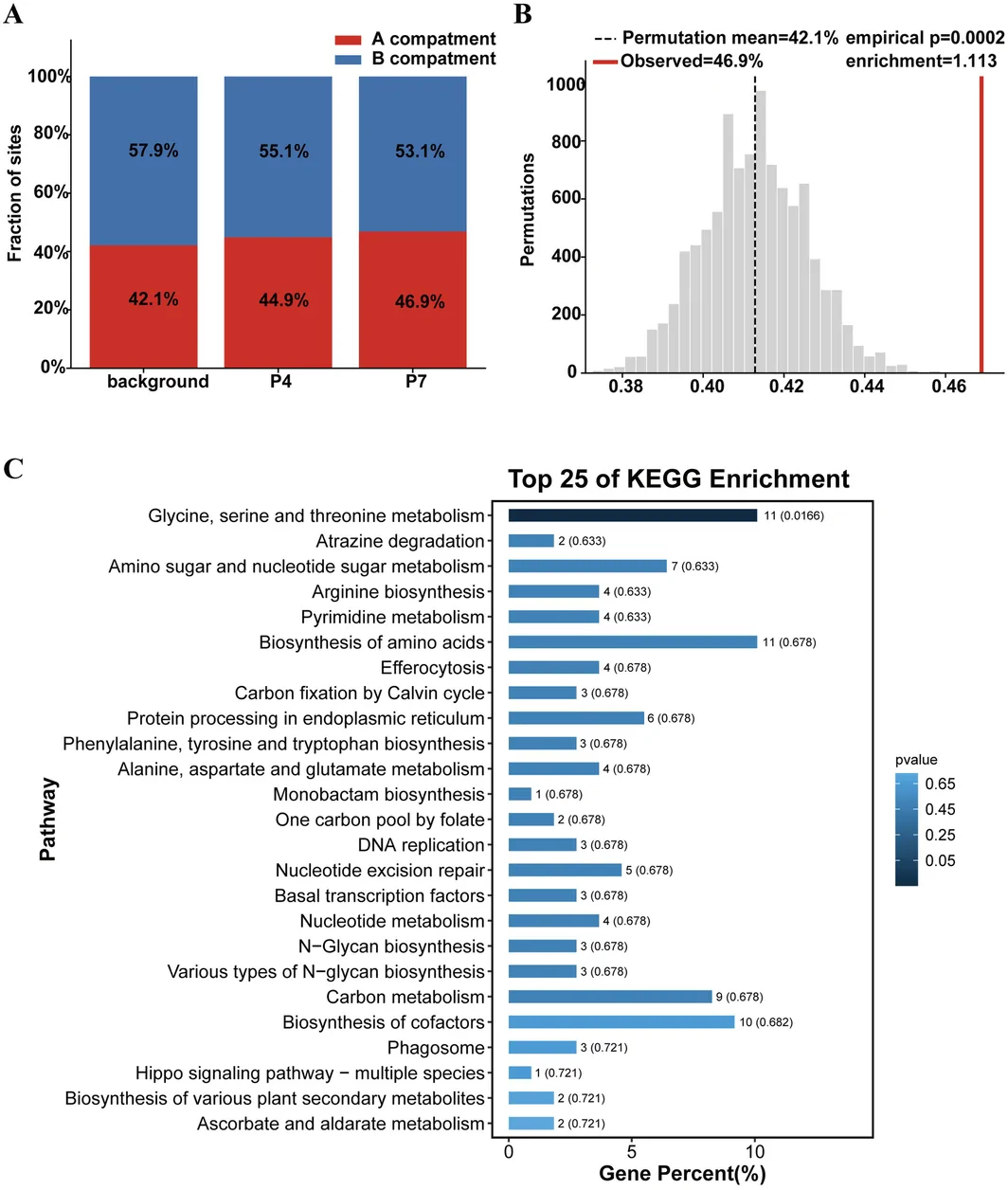
Analysis of the *P. rubens* chromosome architecture and the functional enrichment in P7 library. (A) Distribution of P4 and P7 insertion sites across Hi‐C‐defined compartments A and B. The comparison of P7 sites with the PB insertion background was assessed using a 10,000‐permutation test, and the P4‐versus‐P7 distribution was compared using a χ^2^ test. (B) Permutation‐based enrichment of P7 insertion sites in compartment A. The observed proportion, mean background proportion, enrichment ratio and empirical *p‐*value are indicated. (C) KEGG enrichment analysis of genes associated with P7 insertion sites. The *P. rubens* genome was used as the background gene set, and *p‐*values were adjusted using the Benjamini–Hochberg procedure. Unless otherwise indicated, *n* = 1341 P4 sites, 1222 P7 sites and 13,059 genome‐wide PB insertion sites.

To examine whether P4 and P7 insertion sites preferentially localized at topologically associating domain (TAD) boundaries, we performed permutation tests using the same *PB* background framework. P7 loci showed no significant proximity to TAD boundaries relative to the insertion background, and P4 did not differ from P7 (Figure S5B). In contrast to the compartment effect, P7 sites did not show significant proximity to TAD boundaries, indicating that TAD‐edge localization is unlikely to be a major driver of the observed expression difference.

To characterize the functional categories of genes associated with P4 and P7 insertion loci, KEGG enrichment analysis was performed using the *P. rubens* genome as the background gene set. For the P7 library, genes at or near insertion loci showed significant enrichment in amino acid biosynthesis and carbon metabolism pathways (Figure 4C). The most significantly enriched pathways included glycine, serine and threonine metabolism, indicating that P7‐inserted genes were predominantly involved in growth‐related primary metabolism. In contrast, the P4 library did not show significant metabolic pathway enrichment (Figure S5C). Collectively, these findings indicate that compartment A localization and association with active metabolic genes are correlates of high‐expression P7 sites, while B‐compartment status alone does not account for P4 low‐expression. The absence of significant TAD‐boundary enrichment for P7 sites further implies that large‐scale chromatin compartmentalization, rather than local boundary structure, primarily distinguishes the expression potential of integration loci in *P. rubens* .

### 3.5 Heterologous Biosynthesis of Physcion Based on Position Effect in *P. rubens*

Physcion, a naturally occurring anthraquinone, is produced by several globally recognized herbs, including *Rheum, Polygonum* and other plants (Figure 5A). Notably, physcion exhibits significant efficacy against various plant diseases such as powdery mildew, downy mildew, grey mould and anthrax (Ma et al. 2010). Additionally, it demonstrates low toxicity to humans and animals and is environmentally benign. Consequently, physcion has been registered as a novel plant‐derived fungicide and holds promising potential for application in organic vegetable production. Currently, the biosynthetic pathway of physcion has been fully revealed and refactored in *A. terreus* and *A. nidulans* (Qi et al. 2022; Yao et al. 2023).

**FIGURE 5:**
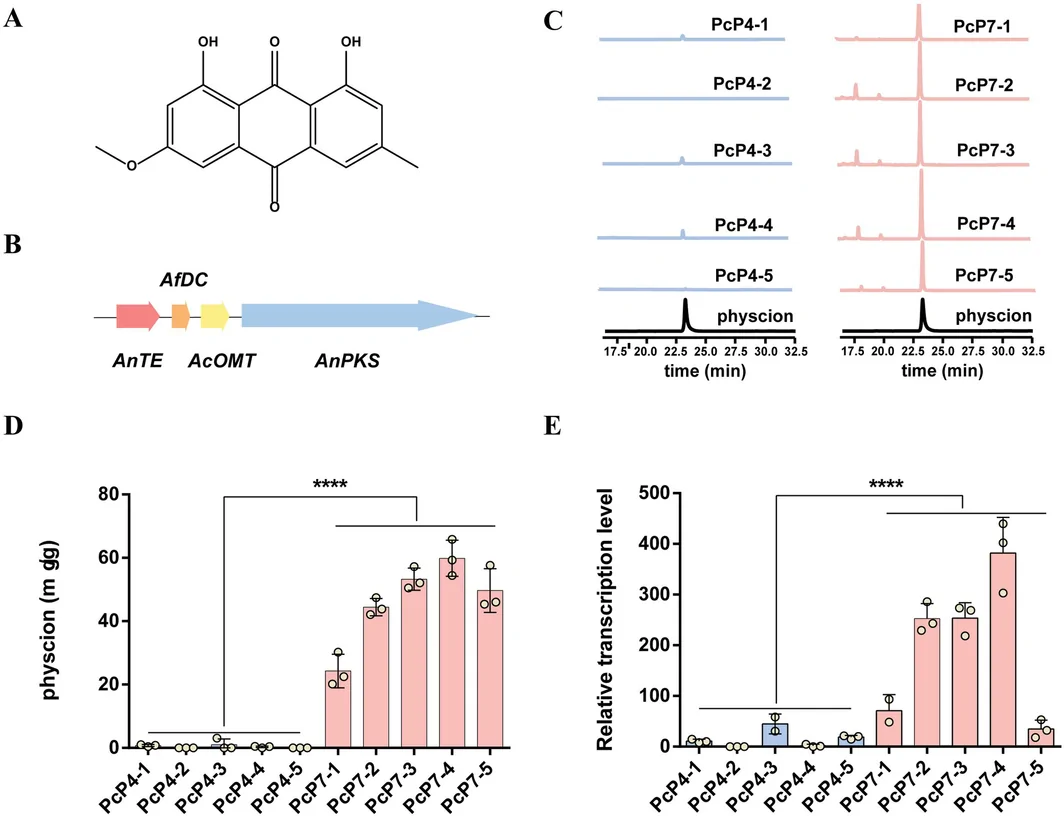
Physcion production of the strains carrying the BGC inserted in the different chromosomal loci of *P. rubens* . (A) Chemical structure of physcion. (B) Organization of the physcion BGC. (C) HPLC analysis of physcion production from the strains carrying the BGC inserted in different chromosomal loci. (D) The production of physcion from the strains carrying the BGC inserted in different chromosomal loci. The average physcion production was approximately 0.95 mg/L for P4‐locus strains and 56.0 mg/L for P7‐locus strains. Physcion concentrations were calculated from an external calibration curve generated using an authentic physcion standard. Values are shown as mean standard deviation from three independent experiments. (E) Relative transcription level of *AnPKS*, a representative gene within the physcion biosynthetic cluster. The relative mRNA expression levels were measured by qRT‐PCR and normalized to the housekeeping gene *β‐Actin*. Values are shown as mean standard deviation from three independent experiments. The results shown are the mean ± S.D. (*n* = 3), based on Student's t test compared to that of PcP4. Statistical significance was determined by two‐way ANOVA followed by Tukey's HSD post hoc test, ****p < 0.0001.

Since penicillin is the primary endogenous secondary metabolite in *P. rubens* , we first disrupted *pcbAB* to eliminate this competitive pathway before introducing the physcion BGC. To ascertain the successful expression of physcion in *P. rubens* , the synthetic BGC of physcion was cloned into the plasmid pAG (Figure 5B). The resulting plasmid designated as pAG‐phy was subsequently introduced into the *pcbAB* disruption mutant (pcbABDM) of *P. rubens* . Fermentation analysis confirmed the physcion production in pcbABDM (Figure S6A, S6B, and S6C). These results demonstrated physcion has been successfully produced in *P. rubens* after introduction of the synthetic physcion BGC.

To demonstrate position effect variegation of *P. rubens* in heterologous expression of secondary metabolites, multiple genomic loci from the P4 and P7 libraries were randomly selected for inserting the physcion BGC (Figure 5B). To exclude potential effects caused by incorrect integration, copy‐number variation or inaccurate quantification, we further verified the engineered strains. Junction PCR confirmed the correct insertion of the physcion BGC at all selected P4 and P7 loci, and qPCR analysis demonstrated single‐copy integration of the BGC (Figure S7A, S7B, S7C, S7D and S7E). The desired product, physcion, was confirmed based on an HPLC retention time identical to that of the authentic standard (Figure 5C). Critically, the strains carrying the BGC inserted in loci from the P4 library produced low levels of physcion, with an average titre of approximately 0.95 mg/L and most samples below 1.2 mg/L. In contrast, strains carrying the BGC inserted in loci from the P7 library produced substantially higher titers, ranging from 27 to 83 mg/L, with an average titre of approximately 56.0 mg/L. Overall, integration into P7 loci resulted in substantially higher physcion production compared with P4 loci. Of them, PcP7‐4 produced the highest yield of physcion. Furthermore, the transcription level of *AnPKS* in these strains was markedly elevated, especially in strains PcP7‐2, PcP7‐3 and PcP7‐4. The higher *AnPKS* transcript abundance observed in several P7 strains was associated with increased physcion production, suggesting that *AnPKS* transcription may contribute to pathway output. (Figure 5D,E). Notably, the discrepancy observed in PcP4‐3 indicates that transcriptional abundance alone may not fully explain the variation in physcion production among different integration loci. Additional regulatory layers associated with chromosomal position may exist, which warrants further investigation.

## 4 Discussion

Chromosomal position effect variegation is widespread in animals, plants and microorganisms. However, position effect in filamentous fungi remains poorly understood due to lacking the suitable tools. In our study, a modified *PB* transposon system was successfully developed and used for investigating the chromosomal position effect in the industrial filamentous fungus *P. rubens*. The observed 8% transposition efficiency, combined with the single‐copy integration characteristic of *PB*, provides a reliable platform for generating insertion mutagenesis libraries. Unlike previous observations in yeast and insect, where TTAA recognition sites dominate, *PB* integration in *P. rubens* showed low positional bias for TTAA recognition sites (Ding et al. 2005; Li et al. 2011). This divergence suggests that *PB* transposition may exhibit species‐specific adaptations, potentially influenced by chromatin accessibility, epigenetic modifications or host factor interactions. The reduced TTAA site preference (40%) raises intriguing questions about fungal genome architecture and the transposase‐DNA interaction dynamics, warranting further structural and biochemical investigations.

Three‐quarters of hyperactive transposases in yeast lacked hyperactivity in the mammalian ES cells, suggesting species‐specific regulation of transposition (Yusa et al. 2011). For example, the *Sleeping Beauty* transposase could enhance transposition through interaction with Miz‐1 transcription factor and HMGB1, while epigenetic modifications may influence transposition frequency (Zayed et al. 2003; Walisko et al. 2006). Reassuringly, unlike certain DNA transposons that cause complex chromosomal rearrangements (Gray 2000; Zhang and Peterson 2005; Geurts et al. 2006), *PB* transposition does not produce such large‐scale genomic changes, and PBase can mediate more precise transposition without compromising genomic integrity (Yusa et al. 2011).

Gene expression levels are regulated by multiple mechanisms, including DNA methylation, epigenetic modifications, three‐dimensional chromatin organization and metabolic network dynamics. In eukaryotic systems, chromosomal position effects have been extensively characterized in *Saccharomyces cerevisiae* , where they are attributed to chromatin compaction states that establish transcriptionally repressive heterochromatin (Grewal and Moazed 2003), the nucleosome structure (Saha et al. 2006) and cis‐regulatory element density near the integration sites (Flagfeldt et al. 2009).

In this study, systematic characterization of the *P. rubens* genomic position effect through GFP‐based screening revealed substantial variability in heterologous gene expression, underscoring the critical role of integration loci in synthetic pathway optimization. Establishment of expression‐inactive loci (P4) and high‐expression loci (P7) libraries provided a valuable resource for the targeted engineering of *P. rubens* , which has been considered as the promising industrial chassis. Heterologous production of physcion demonstrated the practical utility of these libraries for optimizing secondary metabolite biosynthetic pathways.

From a broader perspective, our finding significantly enhanced potential in *P. rubens* as a synthetic biology chassis. The position‐effect mapping strategy could facilitate the engineering of complex biosynthetic pathways in this industrial workhorse. This study not only advanced fundamental understanding of filamentous fungal genome biology but also provided a practical tool for metabolic engineering. The methodologies developed here may serve as a blueprint for genetic manipulation in other industrial fungi, accelerating the development of next‐generation microbial cell factories for pharmaceutical and bio‐based chemical production.

## 5 Conclusion

This study established a *PB*‐based random insertion system for systematically investigating chromosomal position effects in *P. rubens* . Genome‐wide screening identified genomic loci with distinct heterologous expression potentials, and Hi‐C analysis revealed an association between high‐expression P7 loci and chromosome compartment A. Functional validation showed that integration of the physcion biosynthetic gene cluster into P7 loci substantially enhanced physcion production compared with P4 loci, demonstrating a strong influence of chromosomal position on heterologous pathway performance. The discrepancy between *AnPKS* transcript abundance and physcion production further indicates that transcript abundance alone cannot fully explain pathway output. These findings highlight chromosomal position as an important consideration for heterologous pathway engineering in *P. rubens* .

## Author Contributions

**Meng Wu:** methodology, investigation, validation, formal analysis, visualization, writing – original draft. **Yushi Fu:** methodology, data curation, formal analysis. **Linqin Tang:** methodology, data curation, formal analysis. **Yongpeng Yao:** resources, data curation. **Yuanyuan Pan:** data curation, resources. **Gang Liu:** conceptualization, data curation, investigation, validation, writing – review and editing, funding acquisition, formal analysis, supervision, resources, project administration.

## Funding

This work was supported by the National Key Research and Development Program of China, 2021YFA0910600; Strategic Priority Research Program of the Chinese Academy of Sciences, XDA28030000 and National Natural Science Foundation of China, 32570089.

## Ethics Statement

The authors have nothing to report.

## Conflicts of Interest

The authors declare no conflicts of interest.

## Supporting information


**Data S1:** mbt270449‐sup‐0001‐Supinfo.docx.

## Data Availability

The data supporting the findings of this study are available in the manuscript and its Supporting Information file. Chromosome‐level genome assembly was performed using Hi‐C chromatin interaction data. The reference genome sequence can be obtained under accession number JBYQAM000000000. The *PB* transposon sequencing data have been submitted to GenBank SRA (PRJNA1470273, https://www.ncbi.nlm.nih.gov/sra/SRX33598210[accn]). The insertion sites of the genome‐wide *PB* insertions and the insertions identified in the P4 and P7 libraries, respectively, are provided in Datasets S2–S4.
