# Supplementary material for: Position Effect Variegation of Penicillium rubens Genome and Application in Heterologous Production of the Plant‐Derived Anthraquinone Physcion
Source: Microb Biotechnol. 2026 Oct 8;19(10):e70449. doi: 10.1111/1751-7915.70449 (PMC13650407; doi:10.1111/1751-7915.70449)
Supplement: Supplementary file 1 [file MBT2-19-e70449-s001.docx]

Supporting Information

**Position effect variegation of Penicillium rubens genome and application in heterologous production of the plant-derived anthraquinone physcion**

*Meng Wu^1, 2^****,*** *Yushi Fu*^1, 2^, *Linqin Tang*^1, 2^, *Yongpeng Yao^1^, Yuanyuan Pan^1^, Gang Liu^1, 2*^*

^1^State Key Laboratory of Microbial Diversity and Innovative Utilization, Institute of Microbiology, Chinese Academy of Sciences, Beijing 100101, P.R. China

^2^University of Chinese Academy of Sciences, Beijing 100049, P.R. China

*Corresponding author.

Gang Liu: e-mail: [liug@im.ac.cn](mailto:liug@im.ac.cn); Phone: 86-010-64806017

Key words: filamentous fungi, *PiggyBac*, chromosomal position effect, heterologous biosynthesis, physcion

**Figure S1** Amplification of the *PBL* flanking sequences from the different transposable strains by thermal asymmetric interlaced PCR (TAIL-PCR). 1-36, the different transpostion strains; M, 1 kb ladder.

**Figure S2** Genomic distribution of the transposon insertion sites between the intergenic region (IGRs) and the open read frames (ORFs) in the genome-wide insertion library (A), the P4 library (B) and the P7 library (C). In the genome-wide insertion library, the proportion of insertions within ORFs was 35.3%, while the proportion within IGRs was 64.7%. In the P4 library, the proportion of insertions within ORFs was 54.81%, while the proportion within IGRs was 45.19%. In the P7 library, the proportion of insertions within ORFs was 52.86%, while the proportion within IGRs was 47.14%.

**Figure S3** Workflow of single-cell quantification for gene expression in the transposable strains by flow cytometry. During this process, the transposable fungal spores were able to undergo germination, whereas the non-transposable spores were not. The mycelia from the germinated spores were filtered and collected, then they were subjected to an enzymolytic solution and protoplasts were released. However, the enzymolytic solution contained both protoplasts and some unfiltered spores. A trapping solution was introduced to facilitate the separation of spores and protoplasts based on density differences. Consequently, the protoplasts accumulated in the middle layer, while the spores settled at the bottom. The protoplasts were subsequently washed with a 0.7M KCl solution and filtered using a 40 μm filter membrane. Protoplasts derived from the wild-type strain (WT) served as a negative control. Based on variations of the *GFP* expression level, single cell was sorted using flow cytometry.

**Figure S4.** Genetic stability of *PB* insertion copy number and GFP fluorescence across serial passages. A) *PB* copy number in the indicated P4 and P7 strains after passages 1, 3 and 5, as determined by quantitative PCR using *LaeA* as the single-copy reference gene. B) GFP fluorescence intensity of the same strains after passages 1, 3 and 5. Bars represent mean values, error bars indicate standard deviations, and individual dots represent individual measurements. The comparable *PB* copy numbers and GFP fluorescence levels across serial passages indicate that the selected strains maintain stable transgene copy number and reporter expression during propagation. Values are shown as mean standard deviation from three independent experiments. The results shown are the mean ±S.D. (n = 3), based on Student’s t test compared to that of P4-G. Statistical significance was determined by two-way ANOVA followed by Tukey's HSD post-hoc test, ****p < 0.0001.

**Figure S5** Analysis of the *P. chrysogenum* chromosome architecture and the functional enrichment in P4 and P7 libraries. A) Permutation test for P4 enrichment in compartments b(Observed=55.1％, Background mean=57.9％, Enrichment=0.952, empirica p=0.9869). B) Distance to nearest TAD boundary (raw data points). Dashed line = PB-background median (61.62 kb). Boxes show median ± IQR. Wilcoxon two-sided P: P7 vs background = 0.984, P4 vs background = 0.064, P7 vs P4 = 0.161 (BH-FDR 0.984 / 0.318 / 0.402). C) KEGG enrichment of P4 insertion genes.

**Figure S6** Characterization of physcion production in *P. rubens*. A) HPLC chromatogram of physcion in pcbABDM versus engineered strains Pcphy001 at 435 nm. B) UV absorption spectrum of physcion. Physcion in *P. rubens* was characterized by a retention time of 24 minutes with an absorption peak at 435 nm. C) HR-ESI-MS analysis of physcion.

**Figure S7.** Validation of physcion biosynthetic gene cluster integration, copy number, and quantitative assays. A) Verification of the upstream homology-arm junctions of the physcion biosynthetic gene cluster (BGC) integrated into the indicated P4 and P7 strains by junction PCR. Each group of four lanes corresponds to one strain, with the first lane representing the wild-type (WT) control and the second to fourth lanes representing independent transformants. B) Relative copy number of the integrated physcion BGC in the indicated strains, determined by quantitative PCR. C) Standard curve for absolute quantification of *LaeA* copy number, generated using serially diluted templates. The x-axis represents the logarithm of the copy number and the y-axis represents the Ct value; the fitted regression equation is shown on the plot. D) Standard curve for quantitative PCR analysis of the *PKS* gene. The x-axis represents the logarithm of the copy number and the y-axis represents the Ct value; the fitted regression equation is shown on the plot. E) Calibration curve for HPLC-based quantification of physcion using an authentic physcion standard. The x-axis represents physcion concentration (mg/L) and the y-axis represents peak area; the fitted regression equation and coefficient of determination (R²) are shown on the plot.

## Table S1 Strains used in this study.

| **Strains** | **Description** | **Reference** |
| --- | --- | --- |
| *P. rubens* Wisconsin 54-1255 | Wild-type strain (WT) | CGMCC*^a^* |
| Ku70DM | The *ku70* disruption mutant | This study |
| pcbABDM | Disruption of the *pcbAB* in Ku70DM | This study |
| PcWG001 | *pyrG* gene was replaced with a G418 resistance cassette | This study |
| PcWG002 | *PBL-pyrG-PBR* was targeted after the initiation codon ATG of the G418 resistance gene in PcWG001 | This study |
| PcWG003 | *PBL-pyrG-GFP-PBR* was targeted after the initiation codon ATG of *G418* gene in PcWG001 | This study |
| PcWG003P | the plasmid phyPBase-P_glaA_ was transformed into PcWG003 | This study |
| P4-1G | strain within the P4 population isolated by flow cytometry | This study |
| P4-2G | strain within the P4 population isolated by flow cytometry | This study |
| P4-3G | strain within the P4 population isolated by flow cytometry | This study |
| P4-4G | strain within the P4 population isolated by flow cytometry | This study |
| P4-5G | strain within the P4 population isolated by flow cytometry | This study |
| P7-1G | strain within the P7 population isolated by flow cytometry | This study |
| P7-2G | strain within the P7 population isolated by flow cytometry | This study |
| P7-3G | strain within the P7 population isolated by flow cytometry | This study |
| P7-4G | strain within the P7 population isolated by flow cytometry | This study |
| P7-5G | strain within the P7 population isolated by flow cytometry | This study |
| Pcphy001 | the plasmid pAG-Phy was transformed into pcbABDM | This study |
| PcP4-1 | Physcion cassette was targeted to the P4-1 site in pcbABDM | This study |
| PcP4-2 | Physcion cassette was targeted to the P4-2 site in pcbABDM | This study |
| PcP4-3 | Physcion cassette was targeted to the P4-3 site in pcbABDM | This study |
| PcP4-4 | Physcion cassette was targeted to the P4-4 site in pcbABDM | This study |
| PcP4-5 | Physcion cassette was targeted to the P4-5 site in pcbABDM | This study |
| PcP7-1 | Physcion cassette was targeted to the P7-1 site in pcbABDM | This study |
| PcP7-2 | Physcion cassette was targeted to the P7-2 site in pcbABDM | This study |
| PcP7-3 | Physcion cassette was targeted to the P7-3 site in pcbABDM | This study |
| PcP7-4 | Physcion cassette was targeted to the P7-4 site in pcbABDM | This study |
| PcP7-5 | Physcion cassette was targeted to the P7-5 site in pcbABDM | This study |

^a^Chnia General Microbiological Culture Collection Center

## Table S2 Plasmids used in this study.

| **Plasmids** | **Description** | **Reference** |
| --- | --- | --- |
| pUC19 | For genetic manipulation | Novagen |
| pUC19-AMA1 | pUC19 carrying autonomous replication sequences | Laboratory stock |
| pAG | pUC19-AMA1carrying autonomous replication sequences and the G418 resistance gene | This study |
| pACG | pAG carrying the *Cas9* sequences | This study |
| pAZ | carrying autonomous replication sequences and *ZSJS* sequences | This study |
| pACG-pyrg | gRNA for targeted to the 5’ sequence of *pyrG* gene | This study |
| p19-pyrgG418 | The *G418* cassette was targeted to the *pyrG* gene | This study |
| pPB-pyrgG | *PB* and *GFP* cassette targeted to the *G418* gene | This study |
| phyPBase-P_gpdA_ | pAZ carrying the *hyPBase* gene under control of the promoter P_gpdA_ | This study |
| phyPBase-P_glaA_ | pAZ carrying the *hyPBase* gene under control of the promoter P_glaA_ | This study |
| pUC19-LaeA | used for detecting *PB* copy number | This study |
| pUC19-PBL | used for detecting *PB* copy number | This study |
| pACG-ku70 | Containing the gRNA for targeted to the 5’ sequence of *ku70* gene | This study |
| pACG-pcbAB | Containing the gRNA for targeted to the 5’ sequence of *pcbAB* gene | This study |
| pACG-pcbABdonor | Containing the gRNA and donor sequence targeted to the *pcbAB* gene | This study |
| pAG-Phy | pAG carrying the physcion BGC | This study |
| pACG-P4-1 | gRNA for targeted to the P4-1 site in pcbABDM | This study |
| pACG-P4-2 | gRNA for targeted to the P4-2 site in pcbABDM | This study |
| pACG-P4-3 | gRNA for targeted to the P4-3 site in pcbABDM | This study |
| pACG-P4-4 | gRNA for targeted to the P4-4 site in pcbABDM | This study |
| pACG-P4-5 | gRNA for targeted to the P4-5 site in pcbABDM | This study |
| pACG-P7-1 | gRNA for targeted to the P7-1 site in pcbABDM | This study |
| pACG-P7-2 | gRNA for targeted to the P7-2 site in pcbABDM | This study |
| pACG-P7-3 | gRNA for targeted to the P7-3 site in pcbABDM | This study |
| pACG-P7-4 | gRNA for targeted to the P7-4 site in pcbABDM | This study |
| pACG-P7-5 | gRNA for targeted to the P7-5 site in pcbABDM | This study |
| pP4-1P | ZSJS and physcion cassette targeted to the P4-1 site in pcbABDM | This study |
| pP4-2P | ZSJS and physcion cassette targeted to the P4-2 site in pcbABDM | This study |
| pP4-3P | ZSJS and physcion cassette targeted to the P4-3 site in pcbABDM | This study |
| pP4-4P | ZSJS and physcion cassette targeted to the P4-4 site in pcbABDM | This study |
| pP4-5P | ZSJS and physcion cassette targeted to the P4-5 site in pcbABDM | This study |
| pP7-1P | ZSJS and physcion cassette targeted to the P7-1 site in pcbABDM | This study |
| pP7-2P | ZSJS and physcion cassette targeted to the P7-2 site in pcbABDM | This study |
| pP7-3P | ZSJS and physcion cassette targeted to the P7-3 site in pcbABDM | This study |
| pP7-4P | ZSJS and physcion cassette targeted to the P7-4 site in pcbABDM | This study |
| pP7-5P | ZSJS and physcion cassette targeted to the P7-5 site in pcbABDM | This study |

## Table S3 Primers used in this study.

| Primers | Sequence (5’-3’) |
| --- | --- |
| **For construction of the strain PcWG001** | |
| pyrG-Leu-F | ATGGCGAATGGAAATTGTAAGCGTTAATTAAGGTAAGATGGCCGAGTTGGTC |
| pyrG-Leu-R | GCTCTAAAACTCCGCATCTCCGAATGGGCGTGATAAGAGTGGGATTCGAAC |
| gRNA-F | CGCCCATTCGGAGATGCGGAGTTTTAGAGCTAGAAATAGCAAG |
| gRNA-R | CTGCAATGCATGGGATCCCCGGGTTAATAAAAAAGCACCGACTCGGTGCCACTT |
| p063-1U-F | GACGGCCAGTGAATTCGAGCTCGGTACCTTTGTGACTGTGGAGGGTG |
| p56-1U-R | AATGGAAATTGTAAGCGTTATTGTAATTGCCTTCTTGACGTCCA |
| p56-3D-F | TCCGAAGTTCCTATAGATCTTTGCAGTTTGGTGCTTCCTT |
| p063-3D-R | TACGCCAAGCTTGCATGCCTGCAGGTCCGGCTGGGTAAGGTGTTTG |
| p56-2G-F | CGTCAAGAAGGCAATTACAATAACGCTTACAATTTCCATTC |
| p56-2G-R | GTAAGGAAGCACCAAACTGCAAAGATCTATAGGAACTTCGG |
| **For construction of the strain PcWG002** | |
| pyrG-Leu-4R | TCTAAAACTAGACACTCAATCCCCACAGTGATAAGAGTGGGATTCGAAC |
| pyrG-gRNA-4F | CTGTGGGGATTGAGTGTCTAGTTTTAGAGCTAGAAATAGCAAG |
| p58-1-F | CTTCGCAGGAACCCAATCTTCAAAATGGTTAACCCTAGAAAGATAGTCTGCGTA |
| p58-1-R | CATCTTCTGTCGATTAATTAATGACAATGTTCAGTGCAG |
| p58-2(1)-F | TGAACATTGTCATTAATTAATCGACAGAAGATGATATTG |
| p58-2(1)-R | AATTGCGACTTGGAGGACATATGCTTGGGTAGAATAGG |
| p58-2(2)-F | CTATTCTACCCAAGCATATGTCCTCCAAGTCGCAATTG |
| p58-2(2)-R | TTCAGTAACGTTAAGTGGATCCTATTGCGCACCCACGCG |
| p58-2(3)-F | CGCGTGGGTGCGCAATAGGATCCACTTAACGTTACTGAA |
| p58-2(3)-R | ACGGTTCCCACAATGGTTAAAAGAAGGATTACCTCTAA |
| p58-3-F | GTTTAGAGGTAATCCTTCTTTTAACCATTGTGGGAACC |
| p58-3-R | CCTGCGTGCAATCCATCTTGTTCAATGTTAACCCTAGAAAGATAATCATATTG |
| p063-1U-F | GACGGCCAGTGAATTCGAGCTCGGTACCTTTGTGACTGTGGAGGGTG |
| p57-1U-R | CATCTTGTTCAATTTAATTAACATTTTGAAGATTGGGTT |
| p57-2D-F | ATCTTCAAAATGTTAATTAAATTGAACAAGATGGATTG |
| p063-3D-R | TACGCCAAGCTTGCATGCCTGCAGGTCCGGCTGGGTAAGGTGTTTG |
| p043-F | GCCGAGTCTCTGCACTGAACATTGTCATTAATTAATCTTGCGTTACGGGCGTAT |
| p061-1-R | GGCATAAATCGAATGTCCGCTTACTTGTACAGCTCGTCC |
| p061-2-F | TGGACGAGCTGTACAAGTAAGCGGACATTCGATTTATGC |
| p061-2-R | CCTTCAATATCATCTTCTGTCGATTAATGTATTGGGATGAATTTTGT |
| **For construction of the strain ku70DM** | |
| ku70-Leu-1R | TAAAACCCTCGCGCGTGTAGCTATCTTGATAAGAGTGGGATTCGAAC |
| ku70-gRNA-1F | ATCAAGATAGCTACACGCGCGAGGGTTTTAGAGCTAGAAATAGC |
| **For construction of the strain pcbABDM** | |
| pcbAB-Leu-1R | ACGCTCACACGGCGTTCTAGTGATAAGAGTGGGATTCGAAC |
| pcbAB-gRNA-1F | CACTAGAACGCCGTGTGAGCGTGTTTTAGAGCTAGAAATAGC |
| pcbABU-F | GGCGAATGGAAATTGTAAGCGTTAATTAAGCTCAGGGTTTGCACTATC |
| pcbABU-R | CCCTCCGATATGCAACATGGAGGAGAAGATGAAGGTTG |
| pcbABD-F | CTTCATCTTCTCCTCCATGTTGCATATCGGAGGGTT |
| pcbABD-R | GACCAACTCGGCCATCTTACCTTAATCAAATAGCTCACTGGCGTA |
| **TAIL-PCR** | |
| PBL-a1 | CATGCGTCATTTTGACTCACGCGGTCGT |
| PBL-a2 | ACGATGGACTCCAGTCCGGCCCGGGAGCTCCAAGCGGCGACTGAGAT |
| LAD1-1 | ACGATGGACTCCAGAGCGGCCGCVNVNNNGGAA |
| LAD1-3 | ACGATGGACTCCAGAGCGGCCGCVVNVNNNCCAA |
| AC1 | ACGATGGACTCCAGAG |
| **Quantitative PCR** | |
| RT-PBL-F | GACGCATGCATTCTTGAAAT |
| RT-PBL-R | TCACGCGGTCGTTATAGTTC |
| RT-LaeA-F | ctatttccgagagcgtccag |
| RT-LaeA-R | ttcgcgcttgatagatgtgc |
| RT-PKS-F | AATCGCACACCAGCCACT |
| RT-PKS-R | ACAAGCAGCCTGAATTGTGG |
| β-actin-F | GGTATTATGATTGGTATGGGTC |
| β-actin-R | TTCTCACGGTTGAACTTGG |
| **High-throughput sequencing** | |
| N708 | CAAGCAGAAGACGGCATACGAGATCAGAGAGGGTCTCGTGGGCTCGG |
| DM508 | AATGATACGGCGACCACCGAGATCTACACGTACTGACACACTCTTTCCCTACACGACGCTCTTCCGATC-s-T |
| PBLseq | CACGACGCTCTTCCGATCTGCATGCGTCAATTTTACGCAGACTATCTTTCTA |
| illumina-R | CAAGCAGAAGACGGCATACGAGAT |

## Table S4 Characterization of *PBL* tagged sites in the genome of *P. rubens*.

| **Target** | **Number** | **Site** |
| --- | --- | --- |
| 476 bp downstream of PCh04654 | 22 | TTAC |
| 68 bp upstream of PCh06226 | 1 | ATAA |
| 232 bp upstream of PCh11122 | 3 | ATAA |
| 427 bp upstream of PCh04621 | 2 | TTAT |
| 72 bp downstream of PCh04620 | 4 | ATAA |
| 6bp downstream of PCh05392 | 1 | TTAT |
| 158 bp upstream of PCh09794 | 1 | ATAA |
| 762 bp downstream of PCh05621 | 1 | TTAC |
| 1171 bp downstream of PCh04621 | 1 | TTAA |

## Table S5 Statistics of the sequencing data.

| Sample | Number of  raw reads | Number of reads  being mapped | Percentage of  reads being mapped |
| --- | --- | --- | --- |
| total | 30,674,539 | 7,499,594 | 24.45% |
| P4 | 21,333,734 | 6,600,466 | 30.94% |
| P7 | 13,820,652 | 11,562,152 | 83.66% |
